# Do Preoperative Transfusions Impact Prognosis in Moderate to Severe Anaemic Surgical Patients with Colon Cancer?

**DOI:** 10.3390/curroncol28060391

**Published:** 2021-11-12

**Authors:** Nicolò Tamini, Luca Gianotti, Shadya Darwish, Salvatore Petitto, Davide Bernasconi, Massimo Oldani, Fabio Uggeri, Marco Braga, Luca Nespoli

**Affiliations:** 1 Department of Surgery, ASST Monza-San Gerardo Hospital, 20900 Monza, Italy; oldanimassimo@gmail.com; 2 Department of Medicine and Surgery, University of Milano-Bicocca, 20126 Milano, Italy; luca.gianotti@unimib.it (L.G.); s.darwish@campus.unimib.it (S.D.); s.petitto@campus.unimib.it (S.P.); fabio.uggeri@unimib.it (F.U.); marco.braga@unimib.it (M.B.); luca.nespoli@unimib.it (L.N.); 3 Bicocca Bioinformatics Biostatistics and Bioimaging Centre-B4, School of Medicine and Surgery, University of Milano-Bicocca, 20126 Milano, Italy; davide.bernasconi@unimib.it

**Keywords:** anaemia, blood transfusion, colon cancer, colon surgery, oncologic outcomes

## Abstract

(1) Background: Anaemia is a common finding in patients with colon cancer and is commonly corrected by blood transfusion prior to surgery. However, the prognostic role of perioperative transfusions is still debated. The aim of the present study was to investigate the role of preoperative anaemia and preoperative blood transfusion in influencing the prognosis in colon cancer. (2) Patients and Methods: Patients undergoing elective surgery for colon cancer at a tertiary referral university hospital between January 2010 and December 2018 were included in a retrospective review of a prospectively collected database. Univariate and regression analyses were performed to identify the prognostic role of preoperative anaemia and preoperative transfusions in this homogeneous cohort of patients. (3) Results: A total of 780 patients were included in the final analysis. The estimated five-year overall survival rate was significantly worse in the anaemic group (83.8% in non-anaemic patients, 60.6% in mild anaemic patients, 61.3% in moderate anaemic patients and 58.4% in severe anaemic patients; log-rank < 0.001 vs. non-anaemic patients). Anaemic status was found to be an independent adverse prognostic factor (hazard ratio (HR): 1.46; 95% confidence interval (CI): 1.02–2.07) during multivariate analysis. Among moderate to severe anaemic patients, no significant association was found between preoperative transfusions and the risk of mortality or recurrence. (4) Conclusions: Preoperative anaemia, regardless of its severity, and not preoperative blood transfusion, was independently associated with a worse prognosis after surgery in patients with colonic cancer.

## 1. Introduction

Colon cancer is the third-most common cancer in men and the second-most common cancer in women [1].

Meanwhile, anaemia is a common finding in patients with digestive tract tumors, with an incidence approaching 70% during the natural history of colon cancer [2].

The main mechanisms for anaemia include tumour bleeding and reduced iron uptake and use rates [3]. Moreover, anaemia at the time of diagnosis has been associated with a greater risk of tumour recurrence and dismal long-term survival [4,5,6].

Anaemia can be easily corrected by perioperative allogeneic blood transfusions even though their application is thought by some to have immunosuppressive and proinflammatory effects, with possible adverse consequences on postoperative morbidity and long-term prognosis among colon cancer patients [7,8,9].

Conversely, several other studies reported that there was no significant association between blood transfusions and surgery-related complications or overall or disease-free survival after colon cancer resection [10,11,12].

However, preoperative anaemia may lead to transfusion which in turn acts as a mediator in the relationship between anaemia and oncological outcomes.

To date, only a few studies have investigated the independent effects of preoperative anaemia and preoperative blood transfusion on the oncologic prognosis of patients who underwent curative resection for colon cancer [4,9,10].

The aim of the present study was to investigate the correlation between preoperative anaemia and colon cancer prognosis and to clarify the role of preoperative blood transfusion.

## 2. Methods

This study complied with the Strengthening the Reporting of Observational Studies in Epidemiology (STROBE) guidelines [13].

Moreover, the study protocol followed the ethical guidelines of the 1975 Declaration of Helsinki (as revised in Brazil 2013). The local ethical committee review of the protocol deemed that formal approval was not required owing to the retrospective, observational, and anonymous nature of this study.

From 2010 to late 2018, the records of all patients admitted at our institution (Ospedal San Gerardo—Monza, a tertiary referral university hospital in the north of Italy) with a diagnosis of colorectal cancer were collected prospectively. Baseline, surgical, endoscopic, oncological, and surveillance data of all these patients were entered into an electronic database by the medical staff.

Patients who were older than 18 years and submitted to colonic surgery for stage I to IV colon cancer between January 2010 and December 2018 were considered to be eligible for inclusion in this study.

Meanwhile, exclusion criteria included the presence of rectal cancer or recurrent cancer and the performance of emergency surgery or palliative non-resection surgery.

Anaemia was defined according to the World Health Organization’s criteria based on sex (i.e., haemoglobin level ≤13 g/dL in men and ≤12 g/dL in women) [14] and was graded as mild (haemoglobin level of 11 and 12.9 g/dL in men and between 11 and 11.9 g/dL in women), moderate (haemoglobin level between 9 and 10.9 g/dL for both sexes), and severe (haemoglobin level less than 8 g/dL for both sexes).

Preoperative blood transfusion was defined as the receipt of at least one unit of allogeneic packed red blood cells between the time of hospital admission to the start of the surgical procedure.

Allogeneic packed red cell units were made leucocyte-depleted at the local blood bank.

### 2.1. Variables

Preoperative clinical and laboratory data were retrieved from a dedicated institutional database.

The American Society of Anesthesiologists (ASA) score [15] and Charlson Comorbidity Index (CCI) [16] were used to scale surgical risk and comorbid conditions.

The right-side of the colon was considered to extend from the cecum to the transverse colon, while the left-side of the colon was considered to include the splenic flexure to the sigmoid colon.

Pathology reports included tumour classification as defined by the 8th edition of the American Joint Committee on Cancer staging manual [17].

Postoperative complications were considered as any kind of deviation from the normal recovery requiring pharmacological or interventional treatments within 30 days after surgery.

Postoperative 30-day morbidity was graded according to the Clavien–Dindo classification [18].

### 2.2. Surveillance and Survival Assessments

All patients underwent standardised clinical follow-up according to the current international recommendations [19].

The decision as to whether or not to administer adjuvant chemotherapy was made with consideration of the tumour stage, risk factors, and the performance status of each patient. Disease recurrence was assessed by histologic, clinical, and/or radiologic examinations. Recurrence was defined as local, regional, or distant, according to the site of relapse.

Overall survival (OS) was defined as the time interval in months from the occurrence of surgery to death; if a patient was still alive at the end of the study, their data were censored at the last visit available.

Meanwhile disease-free survival (DFS) was defined as the time interval (in months) from surgery to disease recurrence. In the case of no recurrence or death, data were censored at the date of the last available follow-up. When DFS was evaluated, stage IV cancer patients were excluded.

Patient surveillance was stopped at the end of December 2019.

### 2.3. Statistical Analysis

Patients’ characteristics were analysed by descriptive statistics.

For continuous variables, median and interquartile range values were calculated, while for discrete (categorical) variables, the numbers (percentages) falling in each category were recorded.

The normal distribution of continuous variables was assessed by the Kolmogorov–Smirnov test.

Chi-squared tests or Fisher’s exact tests were used to compare categorical variables, while Student’s *t*-tests or Mann–Whitney U tests were performed to compare quantitative variables, as appropriate.

Separately, OS and DFS were evaluated by the Kaplan–Meier method and comparisons among groups were performed with the log-rank test.

Variables potentially associated with survival were analysed by univariate and multivariate Cox regression analyses.

Further, separate regression models including anaemia or blood transfusion were fitted, alongside a model encompassing both variables. Using causal terminology, we can postulate that transfusion is a mediator because it stands on the causal pathway between anaemia and OS but there might be also a direct pathway connecting anaemia to the outcome. Thus, to assess the total effect of anaemia on OS, one should not adjust for transfusion. Conversely, when evaluating the impact of transfusions on OS, anaemia acts as a confounder and should be included in the model as an adjustment factor.

All statistics were two-tailed and significance was accepted when the *p*-value was less than 0.05.

All statistical analyses were performed using the SPSS Statistics version 24.0.0 software program (IBM Corp., Armonk, NY, USA).

## 3. Results

**Figure 1:**
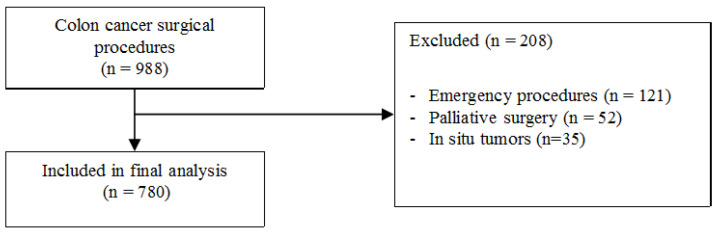
Summary of patients selection.

The details of a total of 988 consecutive patients admitted for colon cancer and submitted to surgery were extracted from a prospectively maintained database. Of these, 121 patients were subsequently excluded due to undergoing emergency procedures, 52 patients were excluded due to receiving palliative surgery without removal of the primary cancer, and 35 patients were excluded because of a diagnosis of carcinoma in situ (Tis). A summary of the patient selection is depicted in Figure 1. Thus, a total 780 patients were included in the final analysis. The characteristics of the study population are shown in Table 1.

Among the study participants, 152 patients (19.5%) presented with mild anaemia at admission, 218 patients (27.9%) presented with moderate anaemia at admission status, and 53 patients (6.8%) presented with severe anaemia at admission.

The preoperative transfusion rate increased according to the grade of anaemia, being 1.3% in patients with mild anaemia, 20.6% in those with moderate anaemia, and 90.6% in those with severe anaemia, respectively.

Anaemia at admittance was significantly more frequent among patients with right-side colon cancer than left-side colon cancer.

Moreover, anaemic patients were generally older and had higher CCI and ASA scores than non-anaemic patients.

Also, increased rates of postoperative blood transfusions and surgical morbidity were observed among anaemic patients when comparing them to non-anaemic patients (24.4% vs. 3.1%; *p* < 0.001 and 31.2% vs. 22.7%; *p* = 0.008, respectively).

The median follow-up period of the study cohort was 42 months (interquartile range: 17–67 months).

**Figure 2:**
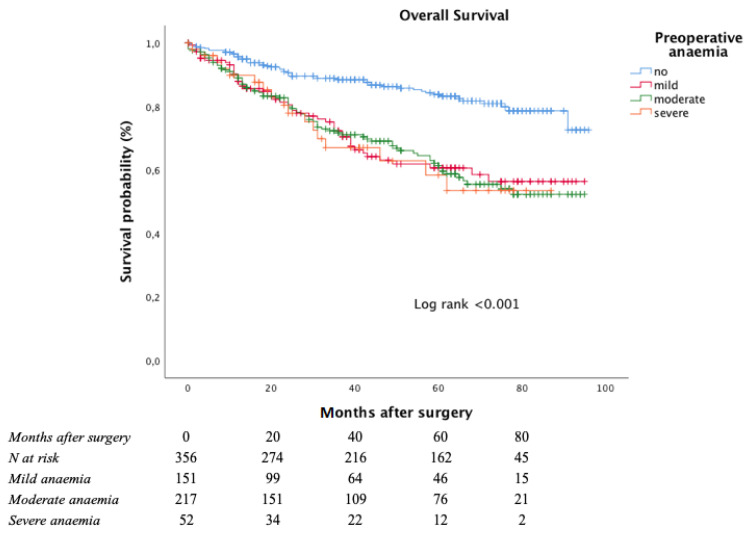
Overall survival according to anaemia severity at admittance.

The Kaplan–Meier curve for OS is shown in Figure 2.

The estimated five-year OS rate was significantly worse among anaemic patients (83.8% in non-anaemic patients, 60.6% in mild anaemic patients, 61.3% in moderate anaemic patients, and 58.4% in severe anaemic patients; log-rank < 0.001 vs. non-anaemic patients).

A multivariate Cox regression analysis was conducted to identify factors associated with OS (Table 2); potential prognostic factors, including CCI score, ASA score, sex, disease stage, tumour differentiation, anaemia at admittance, and preoperative blood transfusion were included in the model.

Together with this analysis, in the regression model, anaemia at admittance and preoperative blood transfusion were also included to evaluate their respective independent roles.

When including both anaemia and blood transfusion in the same model as independent variables, anaemia was found to be an independent prognostic factor for OS (hazard ratio (HR): 1.53, 95% confidence interval (CI): 1.06–2.20) together with advanced cancer stage (HR: 2.35; 95% CI: 1.71–3.23) and CCI score (HR: 1.27; 95% CI: 1.20–1.35). Meanwhile, as depicted in Table 2, blood transfusion was negatively associated with OS in the separate model only (HR: 1.64; 95% CI: 1.10–2.45).

A regression model adjusting for additional clinical variables is depicted in the Supplementary Materials (Table S1).

Moreover, to elucidate the effects of blood transfusions administered prior to the surgical procedure, the subpopulation of moderate and severe anaemic patients (n = 271) was further analysed, since only two transfusional events were present in the remaining proportion of patients.

The baseline characteristics of patients with respect to the degree of preoperative blood transfusion required are reported in Table 3. During univariate analysis, older age, higher ASA score, and lower haemoglobin level at admission were associated with a greater need for a transfusional event.

**Figure 3:**
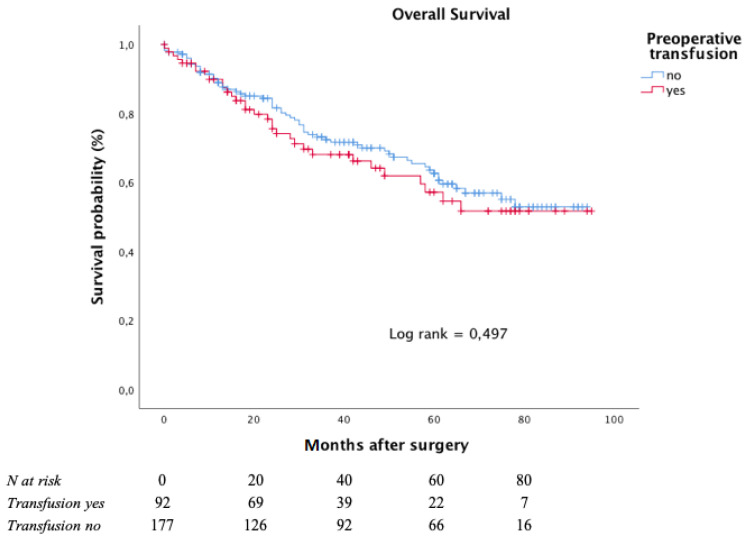
Overall survival according to preoperative transfusion in moderate to severe anaemic patients.

The Kaplan–Meier curves for OS, with respect to preoperative blood transfusion, is depicted in Figure 3.

After a median follow-up of 38 months (interquartile range: 15–64 months), the five-year OS rate was 62.6% in the subgroup of patients who had not undergone any preoperative transfusions and 57.1% in those who had undergone at least one preoperative blood transfusion.

**Figure 4:**
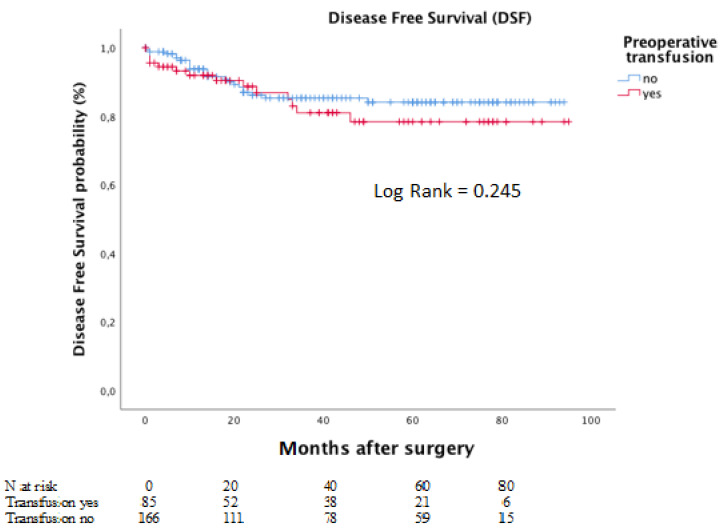
Disease-free survival according to preoperative transfusion in moderate to severe anaemic patients.

Meanwhile, the corresponding five-year DFS rates for the same groups were 78.4% and 84.1%. The Kaplan–Meier curves for DFS is depicted in Figure 4.

During regression analysis (Table 4), a higher stage of disease was associated with both OS (HR: 2.60, 95% CI: 1.60–4.22) and DFS (HR: 4.21, 95% CI: 1.80–9.88), whereas CCI score was correlated with OS only (HR: 1.23, 95% CI: 1.14–1.34).

Ultimately, no significant association was found between preoperative transfusions and the risk of mortality or recurrence.

## 4. Discussion

In the present study, preoperative anaemia, regardless of its severity, was independently associated with worse OS after surgery in patients with colonic cancer; notably, the preoperative use of blood transfusion did not significantly influence this trend.

The negative impact of preoperative anaemia on oncologic outcome among colorectal cancer patients after surgical resection [4,20], or chemotherapy or radiotherapy [21,22], has been described previously.

Different factors may account for these results. In colorectal cancer patients, anaemia is a common preoperative finding, with multiple causative mechanisms. Considering intraluminal tumour bleeding, which frequently occurs in right-sided colon cancer, anaemia may be considered as a feature of more advanced tumours, as it has been associated with cancers that are larger in size and accompanied by higher carcinoembryonic antigen plasma levels [9].

Moreover, an anaemic state has also been associated with a chronic systemic inflammatory response to colorectal cancer and with an impaired nutritional status [5,20], offering an immunopathological basis for the worse oncologic outcome.

Also, anaemia-related hypoxia has been shown to locally exert an immunosuppressive activity at the tumour-microenvironment level, promoting subsequent neoplastic growth [7]; such an increase in postoperative morbidity may, in turn, affect long-term oncologic outcomes.

In our series, a greater prevalence of anaemia at admittance was observed among older and more frail patients and was associated with right-sided colonic cancer, a higher stage of disease, and greater postoperative morbidity; moreover, it was independently associated with impaired OS, confirming previous results [6,12].

In an effort to provide original data to supplement existing knowledge, we focused our analysis on a cohort of patients submitted to colonic surgery only. In fact, rectal cancer patients and those undergoing emergency procedures were excluded to provide a more homogeneous surgical series in terms of surgical trauma, morbidity, and long-term prognosis.

Since intraoperative or postoperative bleeding in elective colonic surgery is often negligible [23], we aimed to provide further evidence of the potential effects of blood transfusion in the preoperative period so as to correctly estimate the risk-to-benefit ratio related to its administration.

Additionally, if preoperative anaemia is hypothesised to negatively impact long-term outcomes, a modulatory effect of its correction might be expected.

Finally, the preoperative period is considered crucial with respect to an immunological response to the surgical trauma in oncologic patients, potentially influencing long-term outcomes.

For these reasons we decided to further analyse the subset of patients presenting with moderate to severe anaemia at admission.

Although possible alternatives exist, such as iron supplementation, allogeneic blood transfusion is currently a widely adopted method to quickly correct anaemia in the preoperative setting.

Transfusion-induced immunomodulation, resulting in an impairment of the immune response, was considered the pathophysiologic base for the association between allogeneic blood transfusion and dismal oncologic or surgical outcomes. However, this association is not fully established given the conflicting results. Most of the current evidence has focused on perioperative blood transfusion, which is often the result of a complex clinical event, encompassing multiple determinants other than the anaemic condition. In such a context, it is difficult to evaluate whether just the blood transfusion itself or other factors necessitating the transfusion influence the prognosis.

Our results, focusing on preoperative transfusional events, suggest that, in moderate and severe anaemic patients, lower levels of haemoglobin at admission as well as a higher ASA score and older age were associated with preoperative blood transfusion. Furthermore, transfusions were not associated with the risk of recurrence or overall mortality, confirming similar previous results [4,5,11,24].

The use, at our hospital institution, of leukocyte-depleted packed red cells across the study period could explain the absence of an impairment in oncologic outcomes, in contrast with the results of other reports [7], presumably because of the reduced occurrence of immune-related transfusion side-effects [25].

Moreover, when considering preoperative anaemia as the surrogate of a biologically more aggressive disease, a clinical effect of its correction should not be expected.

The main limitation of the present study is found with its retrospective design, although the study data were prospectively collected. However, even if a risk adjustment for the observed confounders was performed, potential bias due to unknown confounding factors or selection bias associated with transfusion receipt cannot be excluded.

The monocentric nature of the study can be considered a further weakness of the study despite the large series presented.

Still, notwithstanding these limitations, if considering the ethical issues associated with an ideal randomised trial in this context, results from large cohort analyses may be helpful alternatives for evaluating the effects of blood transfusion on long term outcomes.

## 5. Conclusions

Preoperative anaemia, regardless of its severity, and not preoperative blood transfusion, was independently associated with a worse prognosis after surgery in patients with colonic cancer.

## Figures and Tables

**Table 1:** Baseline characteristics of study population according to anaemia severity.

Baseline Variable		No Anaemia (n = 357)	Mild (n = 152)	Moderate (n = 218)	Severe (n = 53)	* *p* Value
Gender—n (%)	F	167 (46.8)	58 (38.2)	126 (57.8)	33 (62.3)	0.208
	M	190 (53.2)	94 (61.8)	92 (42.2)	20 (37.7)	
Age. Years—median (IQR)		67 (59–76)	75 (67–81)	77 (70–82)	79 (74–83)	<0.001
CCI (Charlson Comorbidity Index)—median (IQR)		5 (4–6)	6 (5–8)	6 (5-8)	6 (5–7)	<0.001
ASA score—n (%)	1	32 (9)	7 (4.6)	6 (2.8)	0 (0)	<0.001
	2	236 (66.3)	66 (43.4)	103 (47.2)	16 (30.2)	
	3	84 (23.6)	74 (48.7)	100 (45.9)	32 (60.4)	
	4	4 (1.1)	5 (3.3)	9 (4.1)	5 (9.4)	
Haemoglobin at admission. g/dL—median (IQR)		13.9 (13.0–15.0)	11.6 (11.0–12.0)	9.8 (9.0–10.0)	7.3 (6.1–7.7)	<0.001
Preoperative transfusion—n (%)		0 (0)	2 (1.3)	45 (20.6)	48 (90.6)	
Blood units transfused—median (IQR)		0 (0)	1.5 (1–2)	2 (1–2)	4 (2–4)	
Preoperative haemoglobin. g/dL—median (IQR)		13.7 (13.0–15.0)	11.5 (11.0–12.0)	10.1 (9.4–10.6)	10.0 (9.4–10.6)	<0.001
Tumour site—n (%)	Right colon	194 (54)	107 (70.4)	169 (77.5)	45 (84.9)	<0.001
	Left colon	163 (45.7)	45 (29.6)	49 (22.5)	8 (15.1)	
Stage—n (%)	1	134 (38.4)	30 (19.7)	33 (15.1)	9 (17)	<0.001
	2	94 (26.3)	52 (34.2)	94 (43.1)	20 (37.7)	
	3	105 (29.4)	53 (34.9)	76 (34.9)	21 (39.6)	
	4	24 (6.7)	17 (11.2)	15 (6.9)	3 (5.7)	
Postoperative transfusion—n (%)		11 (3.1)	17 (11.2)	36 (16.5)	8 (15.1)	<0.001
Postoperative morbidity—n (%)		81 (22.7)	41 (27.0)	71 (32.6)	20 (37.7)	0.008
Postoperative infection—n (%)		51 (14.3)	24 (15.8)	39 (17.9)	9 (17)	0.296
30 days mortality—n (%)		1 (0.3)	1 (0.7)	2 (0.9)	2 (3.8)	0.151
Postoperative length of stay. days—median (IQR)		8 (7–10)	8 (7–10)	9 (7–12)	9 (7–12)	<0.001
Adjuvant chemotherapy—n (%)		133 (37.3)	55 (36.2)	62 (28.4)	13 (24.5)	0.055

* *p* value refers to the comparison between anaemic and non-anaemic patients.

**Table 2:** Multivariate analysis for overall survival in the study population.

Variable	Preoperative Anaemia and Transfusion HR (95% CI)	Blood Transfusion HR (95% CI)
ASA 3–4 (vs. 1–2)	1.42 (1.01–1.99)	1.48 (1.06–2.07)
Female sex	0.83 (0.61–1.13)	0.84 (0.62–1.23)
Stage 3–4 (vs. Stage 1–2)	2.35 (1.71–3.23)	2.35 (1.71–3.23)
Poorly differentiated tumour (G3 vs. G1–2)	1.21 (0.85–1.71)	1.26 (0.89–1.78)
Preoperative transfusion	1.41 (0.93–2.13)	1.64 (1.10–2.45)
CCI (Charlson Comorbidity Index)	1.27 (1.20–1.35)	1.29 (1.23–1.37)
Anaemia at admittance	1.53 (1.06–2.20)	-

**Table 3:** Baseline characteristics of patients with moderate to severe anaemia.

Variable		Preoperative Transfusion		
		No (n = 178)	Yes (n = 93)	*p* Value
Gender—n (%)	F	100 (56.2)	59 (63.4)	0.249
	M	78 (43.8)	34 (36.6)	
Age, years—median (IQR)		76 (68–82)	79 (74–83)	0.022
CCI (Charlson Comorbidity Index) —median (IQR)		6 (5–8)	6 (5–8)	0.456
ASA score—n (%)	1	5 (2.8)	1 (1.1)	<0.001
	2	93 (52.2)	26 (28)	
	3	73 (41)	59 (63.4)	
	4	7 (3.9)	7 (7.5)	
Haemoglobin at admission, g/dL—median (IQR)		9.9 (9.4–10.5)	7.9 (7.3–8.7)	<0.001
Blood units transfused—median (IQR)		0 (0)	2.0 (2–4)	
Preoperative haemoglobin, g/dL—median (IQR)		9.9 (9.2–10.5)	10.2 (9.6–10.7)	0.204
Procedure—n (%)	Right colectomy	138 (77.5)	76 (81.7)	0.659
	Left colectomy	40 (22.5)	17 (18.3)	
Stage—n (%)	1	28 (15.7)	14 (15)	0.991
	2	74 (41.6)	40 (43)	
	3	65 (36.5)	32 (34.4)	
	4	11 (6.2)	7 (7.5)	
Postoperative transfusion—n (%)		30 (16.9)	14 (15.1)	0.703
Postoperative morbidity—n (%)		58 (32.6)	33 (35.5)	0.631
Postoperative infection—n (%)		32 (18)	16 (17.2)	0.874
30 days mortality—n (%)		1 (0.6)	3 (3.2)	0.084
Postoperative length of stay, days—median (IQR)		9 (7–12)	9 (8–13)	<0.001
Adjuvant chemotherapy—n (%)		56 (31.5)	19 (20.4)	0.054

**Table 4:** Multivariate analysis for overall survival (**A**) and disease-free survival (**B**) in patients with moderate to severe anaemia.

(**A**) **OS**				
**Variable**	***p* Value**	**HR**	**HR (95.0% CI)**	
			**Inferior**	**Superior**
ASA 3–4 (vs. 1–2)	0.435	1.23	0.73	2.08
Female sex (vs. male sex)	0.960	0.99	0.64	1.54
Adjuvant chemotherapy	0.439	0.80	0.45	1.42
Postoperative morbidity	0.210	1.33	0.90	2.09
Stage 3–4 (vs. Stage 1–2)	<0.001	2.60	1.60	4.22
Poorly differentiated tumour	0.648	1.13	0.67	1.89
Preoperative transfusion	0.521	1.16	0.73	1.85
CCI (Charlson Comorbidity Index)	<0.001	1.23	1.14	1.33
(**B**) **DFS**				
**Variable**	***p* Value**	**HR**	**HR (95.0% CI)**	
			**Inferior**	**Superior**
ASA 3–4 (vs. 1–2)	0.104	2.31	0.84	6.32
Female sex (vs. male sex)	0.534	1.34	0.53	3.34
Adjuvant chemotherapy	0.382	0.61	0.20	1.84
Postoperative morbidity	0.116	0.42	0.14	1.24
Stage 3 (vs. Stage 1–2)	<0.001	6.38	2.25	18.06
Poorly differentiated tumour	0.541	0.74	0.29	1.92
Preoperative transfusion	0.780	1.14	0.46	2.79
CCI (Charlson Comorbidity Index)	0.386	0.91	0.72	1.13

## Data Availability

Available from the corresponding author upon reasonable request.

## Supplementary Materials

The following are available online at https://www.mdpi.com/article/10.3390/curroncol28060391/s1, Figure S1: Directed Acyclic Graph (DAG) including anaemia, Figure S2: Directed Acyclic Graph (DAG) including preoperative transfusion, Table S1: Modified regression model including postoperative morbidity and adjuvant chemotherapy.
