# Supplementary material for: Do Preoperative Transfusions Impact Prognosis in Moderate to Severe Anaemic Surgical Patients with Colon Cancer?
Source: Curr Oncol. 2021 Nov 12;28(6):4634–44. doi: 10.3390/curroncol28060391 (PMC8628678; doi:10.3390/curroncol28060391)
Supplement: Supplementary file 1 [file curroncol-28-00391-s001.zip › curroncol-1377775-supplementary.pdf]

Supplementary Material

Does preoperative transfusions impact prognosis in moderate to severe anaemic surgical patients with colon cancer?

Nicolò Tamini, Luca Gianotti, Shadya Darwish, Salvatore Petitto, Davide Bernasconi, Massimo Oldani, Fabio Uggeri, Marco Braga and Luca Nespoli

.

•

.

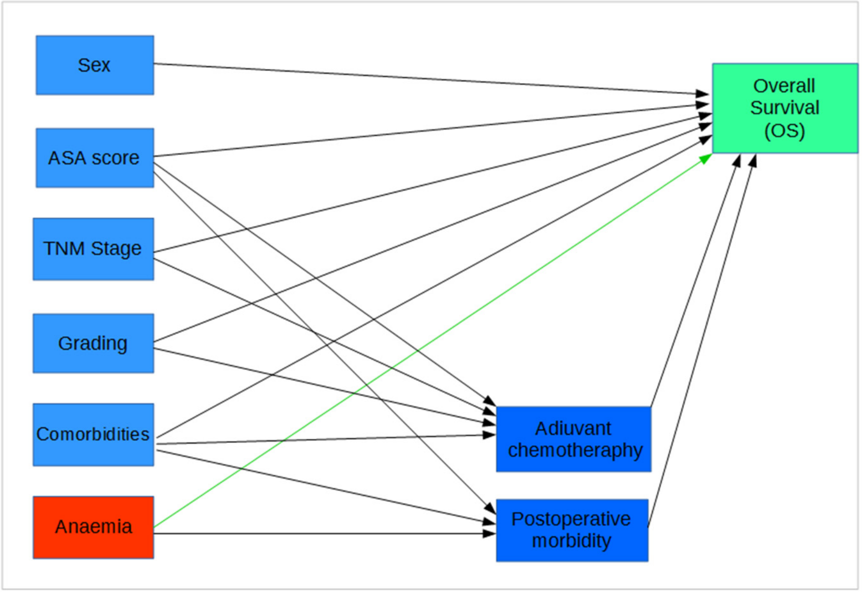

Figure S1. Directed Acyclic Graph (DAG) including anaemia

..

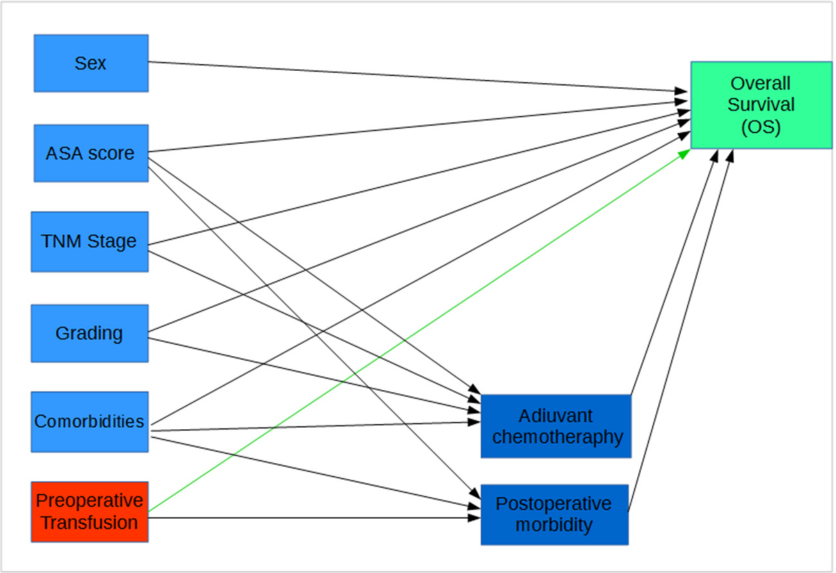

Figure S2: Directed Acyclic Graph (DAG) including preoperative transfusion

Table S1: Modified regression model including postoperative morbidity and adjuvant chemotherapy.

.

.	**Preoperative anaemia and transfusion. HR (95% CI).**	**Preoperative anaemia. HR (95% CI).**	**Blood Transfusion. HR (95% CI)..**
**ASA 3-4 (vs 1-2)**	1.37 (0.97-1.94)	1.40 (0.98-1.98)	**1.43 (1.01-2.03)**
**Female sex**	0.91 (0.68-1.22)	0.92 (0.69-1.24)	0.92 (0.68-1.24)
**Adjuvant chemotherapy**	1.11 (0.78-1.58)	1.08 (0.76-1.53)	1.12 (0.79-1.59)
**Postoperative morbidity**	1.23 (0.90-1.69)	1.25 (0.91-1.71)	1.23 (0.90-1.69)
**Stage 3-4 (vs Stage 1-2)**	**2.30 (1.64-3.23)**	**2.31 (1.65-3.25)**	**2.30 (1.64-3.22)**
**Poorly differentiated tumor (G3 vs G1-2)**	1.20 (0.86-1.68)	1.18 (0.84-1.66)	1.24 (0.89-1.79)
**Preoperative transfusion**	1.33 (0.88-2.01)	-	**1.52 (1.03-2.27)**
**CCI (Charlson Comorbidity Index)**	**1.29 (1.22-1.36)**	**1.28 (1.21-1.35)**	**1.30 (1.23-1.38)**
**Anaemia at admittance**	**1.46 (1.02-2.07)**	**1.55 (1.10-2.17)**	-

Separate regression models including anaemia or blood trasnfusion are fitted, alongside a model encompassing both variables.
